# *rac*-Ethyl 6-hy­droxy-6-methyl-3-oxo-4-phenyl-1,3,4,5,6,7-hexa­hydro­benzo[*c*][1,2]oxazole-5-carboxyl­ate

**DOI:** 10.1107/S1600536811042395

**Published:** 2011-10-22

**Authors:** Arif I. Ismiyev, Abel M. Maharramov, Bahruz A. Rashidov, Gunay Z. Mammadova, Rizvan K. Askerov

**Affiliations:** a Baku State University, Z. Khalilov St. 23, Baku AZ-1148, Azerbaijan

## Abstract

In the title compound, C_17_H_19_NO_5_, the cyclo­hexene ring is in a half-chair conformation and the isoxazole ring in an envelope conformation with the N atom as the flap. The C atoms in the 4- and 6-positions are of the same absolute configuration, whereas the C atom in the 5-position is of the opposite configuration, *i.e.* (4S*,5R*,6S*). The methyl fragment of the eth­oxy­carbonyl group at position 5 is disordered over two sets of sites in a 0.60:0.40 ratio. The crystal packing displays inter­molecular N—H⋯O and O—H⋯O hydrogen bonds.

## Related literature

For general background to the synthesis of isoxazoles, see: Kashima *et al.* (1981 ▶); Goda *et al.* (2003 ▶).
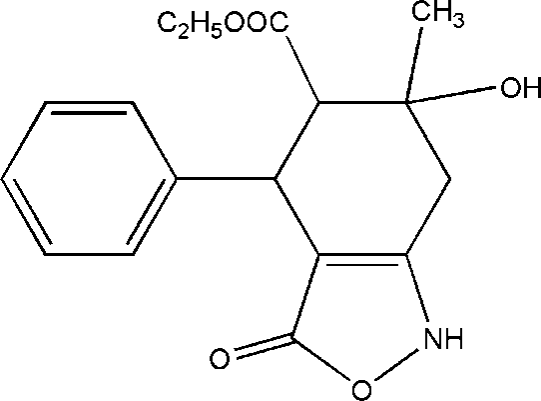


## Experimental

### 

#### Crystal data

C_17_H_19_NO_5_
*M* *_r_* = 317.33
Monoclinic, 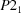

*a* = 6.0712 (6) Å
*b* = 13.4343 (13) Å
*c* = 10.0821 (10) Å
β = 96.882 (2)°
*V* = 816.39 (14) Å^3^
*Z* = 2
Mo *K*α radiation
μ = 0.10 mm^−1^
*T* = 296 K
0.30 × 0.20 × 0.20 mm

#### Data collection

Bruker APEXII CCD diffractometer
Absorption correction: multi-scan (*SADABS*; Sheldrick, 1998 ▶) *T*_min_ = 0.972, *T*_max_ = 0.981
9534 measured reflections
4059 independent reflections
2458 reflections with *I* > 2σ(*I*)
*R*_int_ = 0.049

#### Refinement

*R*[*F* ^2^ > 2σ(*F* ^2^)] = 0.059
*wR*(*F* ^2^) = 0.115
*S* = 1.00
4059 reflections
218 parameters
3 restraints
H atoms treated by a mixture of independent and constrained refinement
Δρ_max_ = 0.17 e Å^−3^
Δρ_min_ = −0.15 e Å^−3^

### 

Data collection: *APEX2* (Bruker, 2005 ▶); cell refinement: *SAINT-Plus* (Bruker, 2001 ▶); data reduction: *SAINT-Plus*; program(s) used to solve structure: *SHELXTL* (Sheldrick, 2008 ▶); program(s) used to refine structure: *SHELXTL*; molecular graphics: *SHELXTL*; software used to prepare material for publication: *SHELXTL*.

## Supplementary Material

Crystal structure: contains datablock(s) global, I. DOI: 10.1107/S1600536811042395/kp2333sup1.cif

Structure factors: contains datablock(s) I. DOI: 10.1107/S1600536811042395/kp2333Isup2.hkl

Supplementary material file. DOI: 10.1107/S1600536811042395/kp2333Isup3.cml

Additional supplementary materials:  crystallographic information; 3D view; checkCIF report

## Figures and Tables

**Table 1:** Hydrogen-bond geometry (Å, °)

*D*—H⋯*A*	*D*—H	H⋯*A*	*D*⋯*A*	*D*—H⋯*A*
N1—H1*A*⋯O6	0.90	1.991	2.846 (3)	159
O6—H6*A*⋯O3	0.82	1.95	2.767 (3)	171

**Fig. 1.**
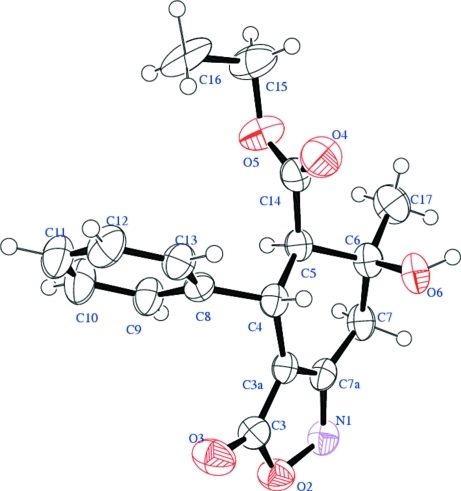
The molecular structure of the title compound with the atomic numbering scheme. Displacement ellipsoids are drawn at the 30% probability level.

**Fig. 2.**
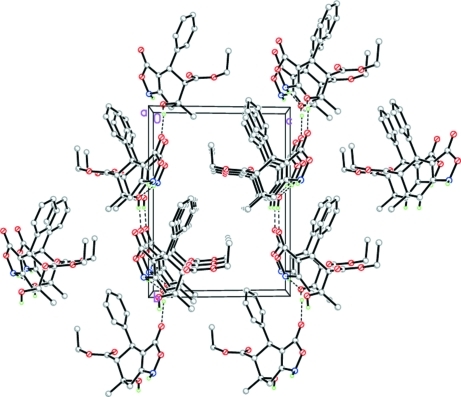
The hydrogen-bonding (dashed lines) in crystal packing of the title compound. H atoms not involved in hydrogen bonding have been omitted for clarity.

## supplementary crystallographic information

### Comment

The wide range of biological activities of isoxazoles has made them popular synthetic targets. Numerous methods for the synthesis of these heterocycles involve approaches based on either intermolecular cycloaddition of 1,3-dipoles to alkynes or condensations of hydroxylamine with β-diketone equivalent with three carbon 1,3-difunctionalized units bearing *sp* or *sp*^2^ carbons, such as propargylic ketones (Kashima *et al.*1981). Synthesis of isoxazole derivatives has been a subject of consistent interest because of the wide applications of such heterocycles in pharmaceutical and agrochemical industry (Goda *et al.* 2003). The structure of ethyl-6-hydroxy-6-methyl-3-oxo-4-phenyl-1,3,4,5,6,7- hexahydrobenzo[*c*]\ isoxazole-5-carboxylate is (I) reported here (Fig. 1). The cyclohexene ring has a half-chair conformation. The phenyl ring is in a pseudo-equatorial position. The torsion angle between the ethoxycarbonyl group and the phenyl substituent C8—C4—C5—C14 is 60.6 (3) which indicates the pseudo-axial location of hydrogen atoms at C4 and C5. The izoxazole ring has an envelope conformation [the torsion angles C7a—N1—O2—C3 is -6.9 (3)° and N1—O2—C3—C3A is 5.2 (3)°]. The title compound (I) is chiral with three stereogenic centres-(4*S**,5*R**,6*S**). The crystal structure involves intermolecular N—H···O and O—H···O hydrogen bonds (Table 1, Fig. 2).

### Experimental

(rac)-Diethyl-4-hydroxy-4-methyl-6-oxo-2-phenyl-1,3-dicarboxylate (20 mmol), hydroxylamine hydrochloride (20 mmol) were dissolved in 20 ml e thanol. Then, 2 drops of H_2_SO_4_ were added and mixture was stirred at 345–350 K for 10 h. After cooling to a room temperature white crystals were obtained. The crystals were filtered off and washed with ethanol. Then, they were dissolved in ethanol (50 ml)and recrystallized to yield colourless block-shaped crystals of the title compound.

### Refinement

The hydrogen atoms of the NH and OH-groups (I) molecule were localized in the difference-Fourier map and included in the refinement with fixed positional and isotropic displacement parameters [*U*_iso_(H) = 1.5*U*_eq_(C) for CH_3_-group and *U*_iso_(H) = 1.2*U*_eq_(N) for amino groups]. The other hydrogen atoms were placed in calculated positions with and refined in the riding model with fixed isotropic displacement parameters [*U*_iso_(H) = 1.2*U*_eq_(C)].

### Crystal data

**Table:**

C_17_H_19_NO_5_	*F*(000) = 336
*M**_r_* = 317.33	*D*_x_ = 1.291 Mg m^−^^3^
Monoclinic, *P*2_1_	Mo *K*α radiation, λ = 0.71073 Å
Hall symbol: P 2yb	Cell parameters from 1319 reflections
*a* = 6.0712 (6) Å	θ = 2.5–21.8°
*b* = 13.4343 (13) Å	µ = 0.10 mm^−^^1^
*c* = 10.0821 (10) Å	*T* = 296 K
β = 96.882 (2)°	Prism, colourless
*V* = 816.39 (14) Å^3^	0.30 × 0.20 × 0.20 mm
*Z* = 2	

### Data collection

**Table:**

Bruker APEXII CCD diffractometer	4059 independent reflections
Radiation source: fine-focus sealed tube	2458 reflections with *I* > 2σ(*I*)
graphite	*R*_int_ = 0.049
phi and ω scans	θ_max_ = 28.4°, θ_min_ = 2.0°
Absorption correction: multi-scan (*SADABS*; Sheldrick, 1998)	*h* = −8→8
*T*_min_ = 0.972, *T*_max_ = 0.981	*k* = −17→17
9534 measured reflections	*l* = −13→13

### Refinement

**Table:**

Refinement on *F*^2^	Primary atom site location: structure-invariant direct methods
Least-squares matrix: full	Secondary atom site location: difference Fourier map
*R*[*F*^2^ > 2σ(*F*^2^)] = 0.059	Hydrogen site location: difference Fourier map
*wR*(*F*^2^) = 0.115	H atoms treated by a mixture of independent and constrained refinement
*S* = 1.00	*w* = 1/[σ^2^(*F*_o_^2^) + (0.0469*P*)^2^] where *P* = (*F*_o_^2^ + 2*F*_c_^2^)/3
4059 reflections	(Δ/σ)_max_ < 0.001
218 parameters	Δρ_max_ = 0.17 e Å^−^^3^
3 restraints	Δρ_min_ = −0.15 e Å^−^^3^

### Special details

**Table:**

Geometry. All e.s.d.'s (except the e.s.d. in the dihedral angle between two l.s. planes) are estimated using the full covariance matrix. The cell e.s.d.'s are taken into account individually in the estimation of e.s.d.'s in distances, angles and torsion angles; correlations between e.s.d.'s in cell parameters are only used when they are defined by crystal symmetry. An approximate (isotropic) treatment of cell e.s.d.'s is used for estimating e.s.d.'s involving l.s. planes.
Refinement. Refinement of *F*^2^ against ALL reflections. The weighted *R*-factor *wR* and goodness of fit *S* are based on *F*^2^, conventional *R*-factors *R* are based on *F*, with *F* set to zero for negative *F*^2^. The threshold expression of *F*^2^ > σ(*F*^2^) is used only for calculating *R*-factors(gt) *etc*. and is not relevant to the choice of reflections for refinement. *R*-factors based on *F*^2^ are statistically about twice as large as those based on *F*, and *R*- factors based on ALL data will be even larger.

### Fractional atomic coordinates and isotropic or equivalent isotropic displacement parameters (Å^2^)

**Table:**

	*x*	*y*	*z*	*U*_iso_*/*U*_eq_	Occ. (<1)
N1	1.0157 (4)	0.39444 (17)	1.0327 (2)	0.0438 (6)	
H1A	1.144 (6)	0.412 (3)	1.008 (4)	0.080 (12)*	
O2	1.0458 (3)	0.29676 (15)	1.08762 (19)	0.0483 (5)	
O3	0.8411 (3)	0.15884 (16)	1.0854 (2)	0.0596 (6)	
O4	0.0789 (3)	0.33341 (17)	0.7160 (2)	0.0632 (6)	
O5	0.2738 (4)	0.33256 (18)	0.5417 (2)	0.0645 (6)	
O6	0.3701 (3)	0.47677 (14)	0.90741 (18)	0.0455 (5)	
H6A	0.3154	0.5327	0.9028	0.068*	
C3	0.8575 (4)	0.2429 (2)	1.0425 (3)	0.0407 (7)	
C3A	0.7212 (4)	0.30374 (18)	0.9509 (2)	0.0328 (6)	
C4	0.5086 (4)	0.27814 (19)	0.8668 (3)	0.0351 (6)	
H4A	0.3867	0.2849	0.9217	0.042*	
C5	0.4751 (4)	0.35572 (19)	0.7527 (3)	0.0354 (6)	
H5A	0.5890	0.3429	0.6937	0.042*	
C6	0.5070 (4)	0.46411 (19)	0.8025 (3)	0.0398 (7)	
C7	0.7502 (4)	0.4776 (2)	0.8598 (3)	0.0419 (6)	
H7A	0.8412	0.4833	0.7874	0.050*	
H7B	0.7665	0.5384	0.9119	0.050*	
C7A	0.8259 (4)	0.39149 (19)	0.9458 (3)	0.0374 (7)	
C8	0.5037 (4)	0.17410 (18)	0.8103 (3)	0.0339 (6)	
C9	0.6769 (5)	0.1395 (2)	0.7449 (3)	0.0464 (7)	
H9A	0.8001	0.1799	0.7398	0.056*	
C10	0.6695 (6)	0.0463 (2)	0.6873 (4)	0.0627 (9)	
H10A	0.7857	0.0248	0.6421	0.075*	
C11	0.4910 (6)	−0.0146 (2)	0.6965 (3)	0.0619 (9)	
H11A	0.4865	−0.0777	0.6584	0.074*	
C12	0.3196 (5)	0.0175 (2)	0.7619 (3)	0.0548 (8)	
H12A	0.1988	−0.0240	0.7687	0.066*	
C13	0.3256 (5)	0.1115 (2)	0.8177 (3)	0.0425 (7)	
H13A	0.2074	0.1329	0.8611	0.051*	
C14	0.2531 (5)	0.3393 (2)	0.6713 (3)	0.0438 (7)	
C15	0.0727 (7)	0.3158 (3)	0.4504 (4)	0.0902 (13)	
H15A	0.0826	0.3450	0.3633	0.108*	
H15B	−0.0578	0.3412	0.4860	0.108*	
C16	0.074 (2)	0.2027 (3)	0.445 (2)	0.110 (4)	0.60
H16A	0.0366	0.1766	0.5284	0.165*	0.60
H16B	0.2192	0.1799	0.4310	0.165*	0.60
H16C	−0.0324	0.1802	0.3736	0.165*	0.60
C16'	−0.009 (4)	0.2095 (5)	0.427 (4)	0.110 (4)	0.40
H16D	0.0820	0.1757	0.3702	0.165*	0.40
H16E	−0.1601	0.2102	0.3862	0.165*	0.40
H16F	−0.0011	0.1753	0.5115	0.165*	0.40
C17	0.4449 (5)	0.5384 (2)	0.6916 (3)	0.0588 (9)	
H17A	0.2885	0.5343	0.6630	0.088*	
H17B	0.5256	0.5237	0.6176	0.088*	
H17C	0.4812	0.6044	0.7237	0.088*	

### Atomic displacement parameters (Å^2^)

**Table:**

	*U*^11^	*U*^22^	*U*^33^	*U*^12^	*U*^13^	*U*^23^
N1	0.0367 (14)	0.0418 (15)	0.0536 (16)	−0.0085 (12)	0.0080 (12)	−0.0056 (12)
O2	0.0403 (10)	0.0500 (13)	0.0525 (12)	−0.0055 (10)	−0.0030 (9)	0.0039 (10)
O3	0.0580 (13)	0.0482 (14)	0.0691 (14)	−0.0091 (10)	−0.0063 (11)	0.0181 (11)
O4	0.0393 (11)	0.0811 (17)	0.0704 (15)	−0.0006 (11)	0.0108 (10)	−0.0120 (13)
O5	0.0652 (13)	0.0827 (17)	0.0436 (12)	−0.0143 (12)	−0.0020 (10)	−0.0021 (12)
O6	0.0438 (10)	0.0340 (10)	0.0620 (13)	0.0023 (9)	0.0194 (10)	−0.0033 (10)
C3	0.0399 (15)	0.0399 (18)	0.0424 (16)	−0.0038 (13)	0.0056 (13)	0.0003 (14)
C3A	0.0347 (13)	0.0279 (13)	0.0367 (14)	−0.0029 (12)	0.0082 (11)	−0.0040 (12)
C4	0.0309 (13)	0.0346 (15)	0.0413 (16)	−0.0029 (11)	0.0100 (12)	−0.0042 (12)
C5	0.0355 (14)	0.0330 (14)	0.0389 (15)	−0.0001 (11)	0.0098 (12)	−0.0003 (11)
C6	0.0436 (15)	0.0329 (15)	0.0450 (17)	−0.0020 (13)	0.0133 (13)	0.0037 (13)
C7	0.0443 (15)	0.0287 (14)	0.0551 (18)	−0.0060 (13)	0.0158 (13)	−0.0020 (13)
C7A	0.0347 (14)	0.0378 (16)	0.0409 (17)	−0.0025 (13)	0.0095 (12)	−0.0096 (13)
C8	0.0325 (14)	0.0347 (15)	0.0340 (15)	−0.0046 (12)	0.0025 (12)	−0.0011 (12)
C9	0.0431 (16)	0.0407 (17)	0.0568 (19)	−0.0052 (13)	0.0119 (15)	−0.0109 (14)
C10	0.061 (2)	0.053 (2)	0.076 (3)	0.0028 (18)	0.0198 (18)	−0.0212 (18)
C11	0.079 (2)	0.0389 (17)	0.066 (2)	−0.0043 (18)	0.0037 (19)	−0.0137 (17)
C12	0.062 (2)	0.0423 (18)	0.060 (2)	−0.0212 (15)	0.0069 (17)	−0.0048 (16)
C13	0.0433 (16)	0.0412 (17)	0.0438 (17)	−0.0043 (13)	0.0083 (13)	−0.0033 (13)
C14	0.0468 (16)	0.0352 (15)	0.0493 (18)	0.0044 (13)	0.0048 (14)	0.0009 (14)
C15	0.095 (3)	0.108 (4)	0.063 (2)	−0.032 (3)	−0.014 (2)	0.002 (2)
C16	0.105 (12)	0.125 (5)	0.087 (7)	−0.055 (4)	−0.043 (9)	−0.008 (4)
C16'	0.105 (12)	0.125 (5)	0.087 (7)	−0.055 (4)	−0.043 (9)	−0.008 (4)
C17	0.067 (2)	0.0434 (18)	0.067 (2)	−0.0003 (15)	0.0113 (17)	0.0132 (16)

### Geometric parameters (Å, °)

**Table:**

N1—C7A	1.361 (3)	C8—C13	1.379 (3)
N1—O2	1.428 (3)	C8—C9	1.387 (4)
N1—H1A	0.88 (3)	C9—C10	1.378 (4)
O2—C3	1.383 (3)	C9—H9A	0.9300
O3—C3	1.218 (3)	C10—C11	1.370 (4)
O4—C14	1.201 (3)	C10—H10A	0.9300
O5—C14	1.330 (3)	C11—C12	1.367 (4)
O5—C15	1.456 (4)	C11—H11A	0.9300
O6—C6	1.432 (3)	C12—C13	1.381 (4)
O6—H6A	0.8200	C12—H12A	0.9300
C3—C3A	1.422 (4)	C13—H13A	0.9300
C3A—C7A	1.343 (3)	C15—C16	1.521 (3)
C3A—C4	1.497 (3)	C15—C16'	1.522 (3)
C4—C8	1.508 (3)	C15—H15A	0.9700
C4—C5	1.548 (3)	C15—H15B	0.9700
C4—H4A	0.9800	C16—H16A	0.9600
C5—C14	1.508 (4)	C16—H16B	0.9600
C5—C6	1.545 (4)	C16—H16C	0.9600
C5—H5A	0.9800	C16'—H16D	0.9600
C6—C17	1.512 (4)	C16'—H16E	0.9600
C6—C7	1.531 (4)	C16'—H16F	0.9600
C7—C7A	1.486 (4)	C17—H17A	0.9600
C7—H7A	0.9700	C17—H17B	0.9600
C7—H7B	0.9700	C17—H17C	0.9600
			
C7A—N1—O2	106.4 (2)	C10—C9—H9A	119.5
C7A—N1—H1A	123 (2)	C8—C9—H9A	119.5
O2—N1—H1A	107 (2)	C11—C10—C9	120.0 (3)
C3—O2—N1	106.91 (19)	C11—C10—H10A	120.0
C14—O5—C15	117.4 (3)	C9—C10—H10A	120.0
C6—O6—H6A	109.5	C12—C11—C10	119.9 (3)
O3—C3—O2	117.9 (2)	C12—C11—H11A	120.0
O3—C3—C3A	134.3 (3)	C10—C11—H11A	120.0
O2—C3—C3A	107.8 (2)	C11—C12—C13	120.0 (3)
C7A—C3A—C3	106.7 (2)	C11—C12—H12A	120.0
C7A—C3A—C4	124.1 (2)	C13—C12—H12A	120.0
C3—C3A—C4	129.1 (2)	C8—C13—C12	121.2 (3)
C3A—C4—C8	113.8 (2)	C8—C13—H13A	119.4
C3A—C4—C5	107.1 (2)	C12—C13—H13A	119.4
C8—C4—C5	110.4 (2)	O4—C14—O5	123.7 (3)
C3A—C4—H4A	108.5	O4—C14—C5	125.1 (3)
C8—C4—H4A	108.5	O5—C14—C5	111.2 (2)
C5—C4—H4A	108.5	O5—C15—C16	99.6 (4)
C14—C5—C6	112.5 (2)	O5—C15—C16'	118.4 (8)
C14—C5—C4	109.6 (2)	C16—C15—C16'	19.9 (11)
C6—C5—C4	113.2 (2)	O5—C15—H15A	111.8
C14—C5—H5A	107.1	C16—C15—H15A	111.8
C6—C5—H5A	107.1	C16'—C15—H15A	107.2
C4—C5—H5A	107.1	O5—C15—H15B	111.8
O6—C6—C17	110.7 (2)	C16—C15—H15B	111.8
O6—C6—C7	109.0 (2)	C16'—C15—H15B	96.9
C17—C6—C7	110.2 (2)	H15A—C15—H15B	109.6
O6—C6—C5	106.90 (19)	C15—C16—H16A	109.5
C17—C6—C5	111.8 (2)	C15—C16—H16B	109.5
C7—C6—C5	108.2 (2)	C15—C16—H16C	109.5
C7A—C7—C6	110.2 (2)	C15—C16'—H16D	109.5
C7A—C7—H7A	109.6	C15—C16'—H16E	109.5
C6—C7—H7A	109.6	H16D—C16'—H16E	109.5
C7A—C7—H7B	109.6	C15—C16'—H16F	109.5
C6—C7—H7B	109.6	H16D—C16'—H16F	109.5
H7A—C7—H7B	108.1	H16E—C16'—H16F	109.5
C3A—C7A—N1	111.7 (2)	C6—C17—H17A	109.5
C3A—C7A—C7	126.2 (2)	C6—C17—H17B	109.5
N1—C7A—C7	122.1 (2)	H17A—C17—H17B	109.5
C13—C8—C9	117.7 (2)	C6—C17—H17C	109.5
C13—C8—C4	121.6 (2)	H17A—C17—H17C	109.5
C9—C8—C4	120.6 (2)	H17B—C17—H17C	109.5
C10—C9—C8	121.1 (3)		
			
C7A—N1—O2—C3	−6.9 (3)	C3—C3A—C7A—C7	177.7 (2)
N1—O2—C3—O3	−174.8 (2)	C4—C3A—C7A—C7	1.1 (4)
N1—O2—C3—C3A	5.2 (3)	O2—N1—C7A—C3A	6.3 (3)
O3—C3—C3A—C7A	178.5 (3)	O2—N1—C7A—C7	−174.5 (2)
O2—C3—C3A—C7A	−1.4 (3)	C6—C7—C7A—C3A	14.4 (4)
O3—C3—C3A—C4	−5.0 (5)	C6—C7—C7A—N1	−164.6 (2)
O2—C3—C3A—C4	175.0 (2)	C3A—C4—C8—C13	132.5 (3)
C7A—C3A—C4—C8	137.7 (2)	C5—C4—C8—C13	−107.1 (3)
C3—C3A—C4—C8	−38.2 (3)	C3A—C4—C8—C9	−49.6 (3)
C7A—C3A—C4—C5	15.5 (3)	C5—C4—C8—C9	70.8 (3)
C3—C3A—C4—C5	−160.4 (2)	C13—C8—C9—C10	1.1 (4)
C3A—C4—C5—C14	−175.1 (2)	C4—C8—C9—C10	−176.9 (3)
C8—C4—C5—C14	60.6 (3)	C8—C9—C10—C11	−1.4 (5)
C3A—C4—C5—C6	−48.6 (3)	C9—C10—C11—C12	0.6 (5)
C8—C4—C5—C6	−172.89 (19)	C10—C11—C12—C13	0.4 (5)
C14—C5—C6—O6	73.4 (3)	C9—C8—C13—C12	0.0 (4)
C4—C5—C6—O6	−51.5 (3)	C4—C8—C13—C12	177.9 (3)
C14—C5—C6—C17	−47.8 (3)	C11—C12—C13—C8	−0.7 (5)
C4—C5—C6—C17	−172.7 (2)	C15—O5—C14—O4	−0.8 (4)
C14—C5—C6—C7	−169.3 (2)	C15—O5—C14—C5	179.5 (3)
C4—C5—C6—C7	65.8 (3)	C6—C5—C14—O4	−74.8 (3)
O6—C6—C7—C7A	71.3 (3)	C4—C5—C14—O4	52.1 (3)
C17—C6—C7—C7A	−167.1 (2)	C6—C5—C14—O5	104.9 (3)
C5—C6—C7—C7A	−44.6 (3)	C4—C5—C14—O5	−128.2 (2)
C3—C3A—C7A—N1	−3.1 (3)	C14—O5—C15—C16	−91.7 (10)
C4—C3A—C7A—N1	−179.8 (2)	C14—O5—C15—C16'	−84.6 (19)

### Hydrogen-bond geometry (Å, °)

**Table:**

*D*—H···*A*	*D*—H	H···*A*	*D*···*A*	*D*—H···*A*
N1—H1A···O6^i^	0.897	1.991	2.846 (3)	158.84
O6—H6A···O3^i^	0.82	1.95	2.767 (3)	171.

Symmetry codes: (i) .
